# High brightness terahertz quantum cascade laser with near-diffraction-limited Gaussian beam

**DOI:** 10.1038/s41377-024-01567-2

**Published:** 2024-08-16

**Authors:** Rusong Li, Yunfei Xu, Shichen Zhang, Yu Ma, Junhong Liu, Binru Zhou, Lijun Wang, Ning Zhuo, Junqi Liu, Jinchuan Zhang, Shenqiang Zhai, Shuman Liu, Fengqi Liu, Quanyong Lu

**Affiliations:** 1 Division of Quantum Materials and Devices, Beijing Academy of Quantum Information Sciences, Beijing, 100193 China; 2 Beijing National Laboratory for Condensed Matter Physics, Institute of Physics, Chinese Academy of Sciences, Beijing, 100190 China; 3 School of Physical Sciences, University of Chinese Academy of Sciences, Beijing, 100049 China; 4 Key Laboratory of Semiconductor Materials Science, Institute of Semiconductors, Chinese Academy of Sciences, Beijing, 100083 China

**Keywords:** Quantum cascade lasers, Terahertz optics

## Abstract

High-power terahertz (THz) quantum cascade laser, as an emerging THz solid-state radiation source, is attracting attention for numerous applications including medicine, sensing, and communication. However, due to the sub-wavelength confinement of the waveguide structure, direct beam brightness upscaling with device area remains elusive due to several mode competition and external optical lens is normally used to enhance the THz beam brightness. Here, we propose a metallic THz photonic crystal resonator with a phase-engineered design for single mode surface emission over a broad area. The quantum cascade surface-emitting laser is capable of delivering an output peak power over 185 mW with a narrow beam divergence of 4.4° × 4.4° at 3.88 THz. A high beam brightness of 1.6 × 10^7 ^W sr^−1^m^−2^ with near-diffraction-limited M^2^ factors of 1.4 in both vertical and lateral directions is achieved from a large device area of 1.6 × 1.6 mm^2^ without using any optical lenses. The adjustable phase shift between the lattices enables a stable and high-intensity surface emission over a broad device area, which makes it an ideal light extractor for large-scale THz emitters. Our research paves the way to high brightness solid-state THz lasers and facilitates new applications in standoff THz imaging, detection, and diagnosis.

## Abstract (web summary)

A high-brightness electrically pumped terahertz quantum cascade surface emitting laser empowered by a phase-engineered photonic crystal cavity.

## Graphical abstract

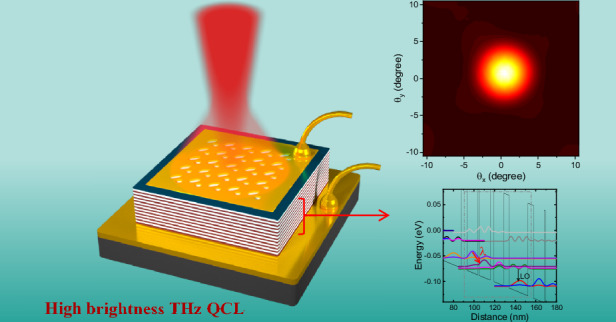

## Introduction

Terahertz (THz) waves (typically 30–3000 μm) are electromagnetic waves between millimeter waves and infrared light, which have important applications in the fields of imaging, sensing, communication, etc^1–3^. THz quantum cascade laser (THz QCL) is a kind of unipolar semiconductor laser source based on intersubband optical transitions and electron resonant tunneling in superlattices or coupled multiple quantum wells^4,5^. It is becoming an attractive laser source for its broad spectral coverage spanning 1.0–5.4 THz, chip-size dimension, electrical pumping, and easy integration enabled by modern semiconductor technology^6–8^. Significant amount of effort has been devoted to elevating the operating temperature, and maximum operating temperature of 261 K has been recently demonstrated^9–11^. On the other hand, THz QCLs with high power with narrow beam divergence are in high demand for applications like standoff THz imaging, sensing, and spectroscopy. A high-power-density laser beam would enable many applications for THz QCLs in non-invasive medical diagnosis and security scanning^12^.

However, high brightness cannot be obtained simply by increasing THz QCL device area without elaborate mode selection mechanisms. Traditional distributed-feedback (DFB) grating can provide mode control only in one dimension, often resulting in an elongated output beam^13^. Third-order Bragg grating or phase-locked array was able to deliver some kind of symmetric output beam, however, the stringent phase locking mechanism makes the fabrication process rather complicated^14,15^. An alternative architecture has been demonstrated by building THz QCLs into a vertical external-cavity surface-emitting laser (VECSEL) scheme^16^. Single mode surface emission with a high beam quality has been demonstrated^17,18^. Nevertheless, the external cavity configuration makes it a non-monolithic solution. Photonic crystal (PC) as an artificial microstructure with periodic or quasi-periodic variation of the refractive index of the medium has been used for single-mode photonic crystal surface emitting lasers (PCSELs)^19–22^ with promising applications in the fields of data communications, sensing, LIDAR, material processing, etc. At present, PCSELs have been also studied in the mid-infrared (Mid-IR)^23–26^ and THz bands^27–29^. However, it is difficult for general single-lattice PCSELs to achieve stable single-mode operation over a broad device area because of multiple modes with similar threshold gains competing with each other, which would result in multimode operation and reduced beam brightness.

Recently, inspired by the concepts of topology in the area of condensed matter, topological photonics has witnessed a rapid development since the introduction of energy-band topology into the field of photonics^30–32^. The discovered topologically protected bulk-state, edge-state, and corner-state robustness over defects or perturbations, has revitalized the photonic designs of semiconductor lasers and promoted the demonstrations of topological lasers in telecom^33,34^ and THz bands^35–37^. While robust single mode operation was observed, high output power with a high beam brightness for the topological THz QCL has yet to be demonstrated for real-world applications. In the meantime, high brightness surface emission with high beam quality in the near-infrared wavelength band has been recently demonstrated with a distributed-Bragg-reflector coupled double-lattice photonic crystal resonator^38–43^, however, the high-beam brightness feature with scalable surface emitting power remains obscure for the lasers in the THz spectral range.

In this work, we propose a metallic THz phase-engineered photonic crystal (PEPC) scheme for high-beam brightness QCLs at ~3.9 THz (see Supplementary Materials). Surface emission output power of 185 mW with a narrow-divergence (~4.4° × 4.4°) symmetric surface-emitting beam is achieved from an emission area of 1.6 × 1.6 mm^2^ based on a PEPC design, which corresponds to a brightness of over 1.6 × 10^7^ Wm^−2^ sr^−1^. It is capable of suppressing the oscillation of the high-order modes with one order of magnitude higher gain margin than conventional single-lattice counterparts, thereby, enabling single mode operation from an emitting area much broader than what was possible before. It should be noted that the high brightness of the surface-emitting THz PEPC QCL is not achieved by using external optics, but rather by its innately high beam quality with narrow divergence angle. Therefore, we believe that the demonstrated electrically pumped surface emitting THz PEPC QCL will become the ideal laser source for next-generation standoff THz applications.

## Results

### THz QCL emission from a PEPC resonator

**Fig. 1:**
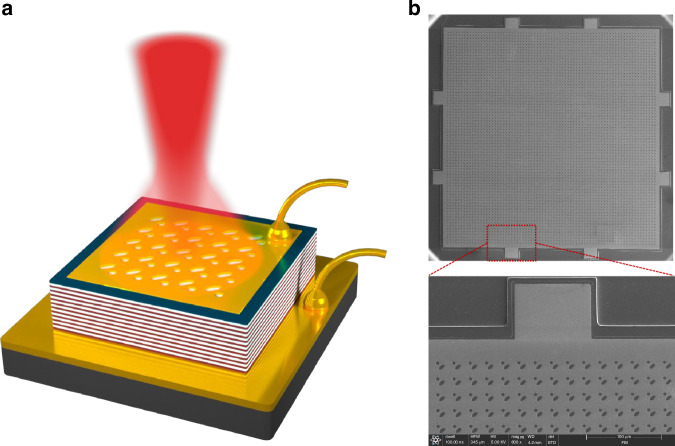
Schematic of an electrically pumped THz PEPC QCL and scanning electron microscope (SEM) images of a PEPC resonator. **a** Schematic diagram of a THz PEPC QCL structure with non-etched active region scheme. The PEPC resonator was constructed by using metal and air interface, adopting a non-etched active region scheme. **b** Top-view SEM images of the fabricated square PEPC resonator (upper panel) and the absorption boundary around an electrode (lower panel). The white bar represents 100 μm. Two spaced electrodes on each side of the device are fabricated for uniform current injection. The outer n+ layer on the square edge of the cavity is retained as the absorption boundary

In this work, to demonstrate the importance of PEPC resonators for high-brightness THz QCL operation, different from the dry-etched active region for most topological surface-emitting lasers and PC QCLs, we adopted a metallic PEPC resonator with a non-etched active region scheme to achieve robust single-mode surface emission. Double-side metal waveguide technology is used to ensure sufficient refractive index contrast. The metallic PEPC resonator consisting of metal and air interface is designed and fabricated on the top metal contact for efficient THz light extraction. Accordingly, we fabricated the device into a square geometry with the designed PEPC lattice patterned on the top surface, as shown in Fig. 1a. The top n+ layer in the air holes is removed by a wet-etching process to reduce the absorption to the surface emission. Top sectional scanning electron microscope (SEM) images of the fabricated PEPC cavity with a lattice constant of *a* = 21.5 μm and phase shift of *d* = 0.3*a* are presented in Fig. 1b. Two electrodes on each side of the square PEPC cavity are fabricated for wire bonding and uniform electrical injection. A 25-μm-wide absorption boundary with top n+ layer unetched is fabricated to increase the optical absorption of the leaky high-order modes, so that the device can work robustly in fundamental mode.

### Computational modeling of THz PEPC resonators

**Fig. 2:**
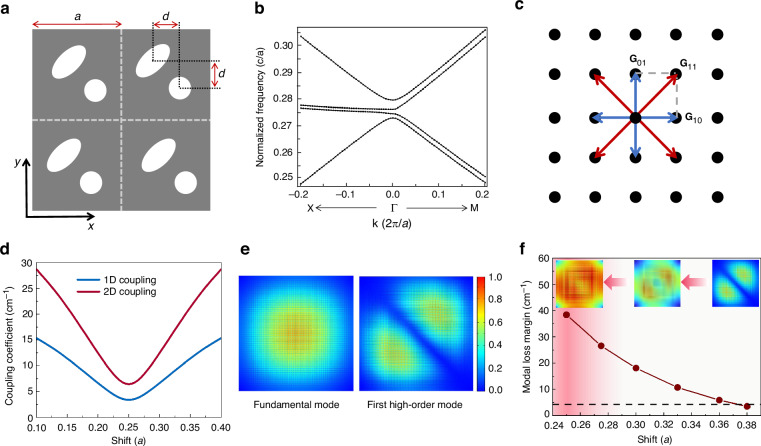
Metallic THz PEPC lattice design and simulation. **a** Schematic of the supercells of the THz PEPC lattice consisting of an elliptical hole (long half axis *a*_1_ = 0.24*a*, short half axis *b*_1_ = 0.12*a*) and a circular hole (radius *r* = 0.12*a*) with a phase shift *d* in both *x* and *y* directions. **b** Calculated photonic band structures of the THz PEPC for TM polarization around the *Γ*_*2*_ point. **c** Schematic of the reciprocal lattice of PEPC and its in-plane coupling diagram. **d** Calculated in-plane 1D and 2D coupling coefficients as a function of the phase shift *d* between the two lattices. **e** Electric field (|*E*_*z*_|^2^) distribution of the fundamental and first high-order modes of the THz PEPC QCL. **f** Modal loss margin between the fundamental and first high-order modes in the PEPC resonator as a function of phase shift. The inset shows the high-order mode profile involving with the phase shift. The dashed line indicates the modal loss margin of a PC cavity with similar lattice parameters. The shaded area indicates the phase shift region where the in-plane 1D coupling strength is insufficient for the given device area

The electrically pumped THz PEPC QCL wafer provides a peak gain at ~3.9 THz (see Supplementary Materials). The phase-engineered photonic crystal supercell is composed of elliptical and circular unit cells with a lattice constant of *a* and a phase shift of *d* along the *x* and *y* directions between the two cells, as shown in Fig. 2a. We calculate the transverse-magnetic (TM) band structure of the PEPC around the second-order *Γ*-point (*Γ*_*2*_) as shown in Fig. 2b. As a result of the couplings among the fundamental Bloch waves, broad area two-dimensional (2D) resonance is formed for surface emission. With the introduction of another set of lattice, the in-plane one-dimensional (1D) couplings between reciprocal lattice vectors **G**_10_ and **G**_-10_, and **G**_01_ and **G**_0-1_ (Fig. 2c) are greatly reduced while the 2D couplings, like the coupling between **G**_10_ and **G**_01_, and **G**_-10_ and **G**_0-1_ are maintained, as the calculated coupling coefficients shown in Fig. 2d. This will expand the mode field throughout the entire cavity area and enhance the in-plane optical loss margin between the fundamental and high-order cavity modes. Figure 2e shows the calculated electric field distributions (|*E*_*z*_|^2^) of the fundamental mode and first high-order mode for a metallic PEPC resonator with cavity side length *L* = 1.6 mm and *d* = 0.3*a*. Due to the mode expanding induced by the phase design of the PEPC, the anti-node of the high-order mode is pushed towards the edges of the cavity, resulting in much higher loss for the high-order mode than that of the fundamental mode. Figure 2f shows the change of modal loss margin Δ*α* of PEPC cavity by sweeping the shift *d* from 0.38*a* to 0.25*a* using the three-dimensional full wave module via finite element method based on a commercially available software. Here, Δ*α* is the cavity loss difference of the fundamental mode and first higher-order mode. In the simulation, the loss is obtained from: $\alpha =2\pi /({aQ})$, where *Q* is the quality factor of the PEPC resonator. When the phase shift *d* decreases to 0.25*a*, the modal loss margin increases significantly by one order of magnitude compared to that of a single-lattice PC cavity with the same lattice parameters (see Supplementary for the PC cavity design). This is crucial to stable single mode operation over a large area. On the other hand, when the phase shift approaching 0.25*a*, while stable high-order modes cannot be formed due to insufficiency of in-plane 1D coupling strength (left inset of Fig. 2f), the optical loss of the fundamental mode also rises up rapidly. As a result, a moderate phase shift *d* = 0.3*a* is chosen for the device area of 1.6 × 1.6 mm^2^ (also see section 5 in Supplementary Materials).

### Characterizations of THz PEPC QCLs

**Fig. 3:**
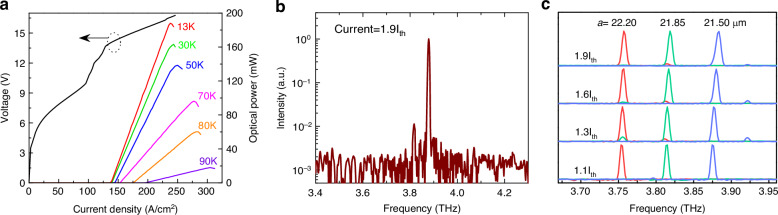
L-I-V and spectral characterizations for THz PEPC QCLs. **a** L-I-V characterization of a THz PEPC QCL with *d* = 0.3*a* and L = 1.6 mm at different temperatures under a pulsed condition with a repetition rate of 10 kHz and a wide pulse of 4-μs duration. The I–V curve was taken at 13 K. **b** Spectrum of THz PEPC QCL (*d* = 0.3*a*) near the roll-over current at 13 K, and the measured SMSR is over 20 dB. **c** Measured normalized lasing spectra of THz PEPC QCLs (*d* = 0.3*a*) with different lattice constants (*a* = 22.20, 21.85, and 21.50 μm) at different bias conditions at 13 K as functions of frequency

The experimental light-current-voltage (L-I-V) curves of a THz PEPC surface emitting QCL with *d* = 0.3*a* and *L* = 1.6 mm are plotted in Fig. 3a. An electrical pulse generator was employed to pump the PEPC QCL in pulsed mode operation with a repetition rate of 10 kHz and a wide pulse of 4*-*μs duration. A low threshold current density of 136 kA/cm^2^ is obtained, which is equivalent to that of a standard second-order DFB THz QCL fabricated from the same wafer. A high peak optical power output up to 185 mW with a slope-efficiency of 90 mW/A was directly measured from a THz PEPC QCL operating at 13 K using a Thomas Keating (TK) terahertz absolute power meter without any collection efficiency correction. The device emits up 90 K and decent power of 60 mW at 80 K is observed owing to the rather low-threshold feature of the quantum design. Since the slope efficiency is determined as: ${\eta }_{s}=(N\hslash \omega {\eta }_{i}{\alpha }_{\perp })/[e({\alpha }_{i}+{\alpha }_{//}+{\alpha }_{\perp })]$, where *N* is the number of QCL stages, $\hslash$ is reduced Planck constant, $\omega$ is the angular frequency, ${\eta }_{i}$ is the internal quantum efficiency, *e* is the electron charge, ${\alpha }_{i}$ is the waveguide loss, ${\alpha }_{//}$ is the in-plane loss, and ${\alpha }_{\perp }$ is the vertical loss. Given a 1.7-mm long and 80-μm wide Fabry–Pérot (FP) device with a slope efficiency of 35 mW/A, using a calculated in-plane losses of 1.7 and 2.5 cm^−1^ for the FP and PEPC devices, a waveguide loss of 10 cm^−1^, and vertical loss ${\alpha }_{\perp }=0$ for FP device, the vertical loss of the PEPC QCL is estimated to be 6.5 cm^−1^, which matches well to that numerical simulation results (see Supplementary Materials). Compared with the topological surface emitting QCLs^36,37^, the output power of PEPC QCL device increases by an order of magnitude. The improvement stems from the stable 2D mode control over a broad area enabled by the proposed THz PEPC lattice design.

Devices with the same area but different shifts are fabricated and tested for comparison (see Supplementary Figure S5). The power peaks at *d* = 0.3*a* and decreases towards 0.25*a* due to insufficient in-plane feedback and thus increased in-plane loss. As a result, to further increase the vertical output power, the key is to make *d* as close as 0.25*a* to evenly spread out the mode distribution over a sufficiently large device area, e.g., 4 × 4 mm^2^, to reduce in-plane loss and improve the vertical radiation efficiency for the fundamental mode and maintain a sufficient loss margin over high-order modes, a high power over 5 W is projected.

The electrically pumped PEPC QCL spectra were measured by a Fourier transform infrared spectrometer (FTIR, Bruker VERTEX 80 v) with a DTGS detector, and the spectral resolution is 0.2 cm^−1^. The single-mode QCL performance is evaluated by the side-mode suppression ratio (SMSR) when the THz PEPC QCL output power is maximum. The SMSR under a bias current (1.9I_th_) near the roll over is above 20 dB for the THz PEPC QCL device, as shown in Fig. 3b. With increasing of the injected current, single-mode operation is maintained within the entire dynamic range of current. The lasing frequency is at ~3.88 THz, very close to the predicted fundamental mode frequency of the PEPC resonator through simulations. Figure 3c shows the measured normalized lasing spectra of THz PEPC QCLs with different lattice constants (*a* = 22.20, 21.85, and 21.50 μm) at different bias currents. The THz PEPC QCLs maintain robust single-mode operation under different bias conditions from near threshold to peak bias. The spectral test results indicate that the device operates stably on fundamental mode under electrical pumping. With increasing bias currents, a slight blueshift of the laser spectral peak is observed due to Stark effect of the intersubband transition with the applied electrical field in the THz PEPC QCL gain medium.

**Fig. 4:**
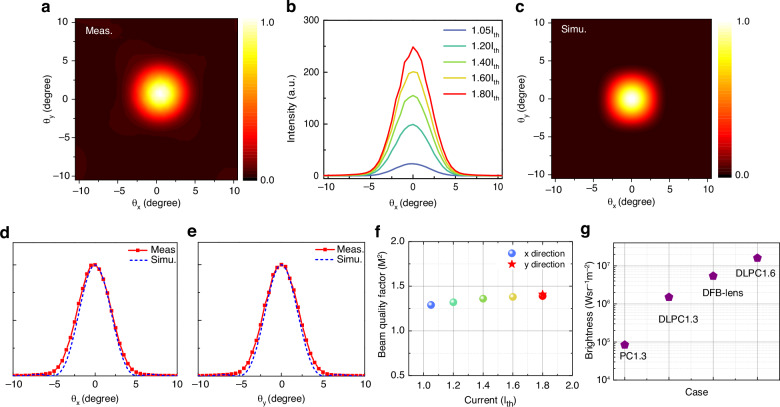
Measured and simulated far-field emission patterns of THz PEPC QCLs. **a** Experimental 2D far-field emission pattern of the THz PEPC QCL, where *θ*_*x*_ and *θ*_*y*_ are angles with respect to the surface normal along the longitudinal and lateral directions of the PEPC, respectively. **b** Experimental 1D far-field distributions of the THz PEPC QCL plotted along *θ*_*x*_ at different bias conditions. For the experimental results, the far field was measured at 13 K with pulse condition 1% (10 kHz, 4 μs). **c** The calculated 2D far-field pattern of the fundamental mode of the THz PEPC cavity. **d**, **e** Experimental and simulated 1D far-field emission patterns plotted along *θ*_*x*_ and *θ*_*y*_, respectively. It can be seen that the simulation results are highly consistent with the experimental results. **f** Beam quality factor (M^2^) values for the THz PEPC QCL at different bias currents. **g** Brightness upscaling of THz PEPC QCL with dimension and brightness comparison with a PC QCL and a DFB QCL with a focal lens from ref. ^45^

The measured 2D far-field emission pattern of the PEPC QCL with *d* = 0.3*a* at a bias current of 1.8I_th_ is presented in Fig. 4a. Near perfect single-lobed Gaussian beam pattern is observed with a narrow beam divergence angle of 4.4° in both vertical and horizontal directions, as shown in Fig. 4d, e. The measured beam quality is near-diffraction-limited when comparing with an ideal Gaussian beam assuming a perfect near field distribution as plotted in Fig. 4c. The more important part is that stable single-lobed far-field patterns across the working dynamic range are observed for the PEPC QCL device, as shown in Fig. 4b. The beam quality factor *M*^2^ is calculated according to ${M}^{2}=4\pi {\sigma }_{0}{\sigma }_{\theta }/\lambda$, where ${\sigma }_{0}$ and ${\sigma }_{\theta }$ are the standard deviations of the calculated near field profile of the fundamental mode for a 1.6 × 1.6 mm^2^ cavity and the measured far-field profile, respectively^44^. Figure 4f shows the *M*^2^ values at different bias voltages for the THz PEPC QCL. At a driving current of 1.8I_th_, a beam quality factor of 1.4 is obtained with a FWHM angle of 4.4° in both directions. Considering the output power of 185 mW at this current, a high beam brightness of *B* = 1.6 × 10^7 ^W sr^−1^ m^−2^ is estimated using $B=P/({\lambda }^{2}{M}_{x}^{2}{M}_{y}^{2})$. Brightness upscaling is also observed by comparing PEPC QCLs with different cavity areas. While the traditional PC QCL shows typical multi-lobed far field due to smaller modal loss margin with *M*^2^ ~ 10, the 1.3 × 1.3 mm^2^ PEPC device exhibits primarily single-lobed far-field distribution with *M*^2^ ~ 3.5 (see Supplementary Materials). As a result, the brightness of the 1.6 × 1.6 mm^2^ PEPC is about two orders of magnitude higher than those from a standard PC QCL and three times higher than a DFB QCL reported in ref. ^45^ after using a focal lens, as summarized in Fig. 4g, marking the brightest THz QCL emitting a direct Gaussian beam to date. Given the above predicted power level for a device with *d* ~ 0.25*a*, a high THz brightness up to GW sr^−1^ m^−2^ level is achievable with a near-diffraction-limited THz beam quality once comprehensive optimizations, including additional loss coupling mechanism, enhanced absorption boundary, and improved thermal-dissipation packaging like Au–Au bonding onto a diamond submount, are performed to maintain the decent modal-loss margins for larger-area devices.

## Discussion

Conventional high-power QCLs inevitably suffer from poor beam quality owing to the onset of many-mode oscillation. In this work, we surmount this challenge by developing large-scale THz PEPC surface-emitting QCLs with controlled modal loss and coupling inside the cavity for stable single-mode surface emission over a broad QCL area. The quantum cascade surface-emitting laser is capable of delivering an output peak power over 185 mW with a narrow beam divergence of 4.4° × 4.4° at 3.88 THz. A stable beam quality factor *M*^2^ of 1.4 in both directions over the dynamic range with a high beam brightness of 1.6 × 10^7 ^W sr^−1^ m^−2^ without using any external optical lenses is achieved from a large device area of 1.6 × 1.6 mm^2^, making it the brightest single-mode surface-emitting single-chip THz QCL capable of direct near-diffraction-limited Gaussian beam emission. More importantly, the power of the THz PEPC QCL can be further upscaled with the device size under a proper shift *d* design. Given the stable single-mode feature of THz PEPC design enabled by the superior modal loss margin between fundamental and higher order modes, a high beam brightness up to GW sr^−1^ m^−2^ level is achievable in the near future, which would open up many new high brightness THz applications. Our work demonstrates a practical approach to brightness enhancement of electrically pumped THz QCL without using any external optical setup. The high-brightness surface-emitting THz PEPC QCLs would open up many new applications in standoff THz imaging, detection, and diagnosis.

## Materials and methods

### Device fabrication

The THz QCL chip is based on a four-well GaAs/AlGaAs hibrid quantum design with a gain pick around 3.9 THz and a active region thickness of 12.15 μm. The fabrication of THz PEPC QCL starts from the In-Au thermocompression wafer bonding. The semi-insulating GaAs substrate of the original wafer was then removed by lapping and selective wet etching. The lattice patterns were defined by an image-reversal lithography followed by a sequence of Ti/Au (40/300 nm) metal deposition and lift-off process as top metallic layers. The 150-nm thick highly doped GaAs contact layer in the unit cells of PEPC lattice is etched away by H_3_PO_4_:H_2_O_2_:H_2_O wet etchant in 1:1:10 concentration to reduce the absorption of the output light. When considering the absorption boundary condition, the high doped absorption layer of the 25-μm-wide boundary of the device is retained. 1 μm silicon oxide was deposited as the hard mask to of mesa etching. Then, the THz PEPC cavity mesa is dry etched 5-μm deep into the active region to minimize any optical feedback from the edge of the cavity. Two spaced rectangular electrodes with size of 100 × 100 μm^2^ at the four edges of the devices were defined for wire bonding and uniform current injection.

### Characterization

The laser chip was cleaved and indium soldered onto a copper heat sink and then mounted in a liquid-helium cryostat for characterization. The output power of the PEPC QCL was directly measured by Thomas Keating (TK) THz absolute power meter without any corrections or focusing optics. The THz spectra were measured using a Fourier transform infrared spectrometer (Bruker VERTEX 80 v) with a DTGS detector, and the resolution is 0.2 cm^−1^. For the L-I-V, spectrum, and far-field emission pattern measurements, the PEPC QCL was driven with a 4-μs current pulse at 4% duty cycle at different testing temperatures. The far-field patterns were measured with a high-sensitivity Golay cell detector scanned on the curve of a sphere with radius around 15 cm from the device. The high-frequency pulse is converted into a low-frequency (20 Hz) signal using a signal generator, and the light intensity detected by the Golay cell detector was characterized with voltage output of a lock-in amplifier.

### Simulation

All of the 3D full-wave simulations and 2D simulations were carried out by using COMSOL Multiphysics 6.0. A module of Electromagnetic waves, Frequency Domain (ewfd) under the catalog of Optics was utilized to calculate the band structure, effective refractive index, eigenmode, modal loss, near field, and far field of a PEPC QCL and a PC QCL for comparison. In the simulation, the refractive index of the metal is 188 + 288i and the thickness of the upper Au layer is 300 nm. A highly doped contact layer serving as absorbing boundaries with refractive index of 4.6865 + 20.2949i calculated by the Drude-Lorenz model. The phase-engineered photonic-crystal pattern was generated with the Klayout Editor Software and imported into the simulation software.

### Supplementary information

Supplementary Information for High brightness terahertz quantum cascade laser with near-diffraction-limited Gaussian beam

## Data Availability

All data in the paper are present in the main text and/or the Supplementary Materials, which will also be provided from the corresponding author upon reasonable request.
