# Supplementary material for: High brightness terahertz quantum cascade laser with near-diffraction-limited Gaussian beam
Source: Light Sci Appl. 2024 Aug 16;13:193. doi: 10.1038/s41377-024-01567-2 (PMC11329767; doi:10.1038/s41377-024-01567-2)
Supplement: Supplementary file 1 — Supplementary Information for High brightness terahertz quantum cascade laser with near-diffraction-limited Gaussian beam [file 41377_2024_1567_MOESM1_ESM.docx]

**Supplementary Information**

**High brightness terahertz quantum cascade laser with near-diffraction-limited Gaussian beam**

Rusong Li^1,2,3,5^, Yunfei Xu^4,5^, Shichen Zhang^1,5^, Yu Ma^1^, Junhong Liu^4^, Binru Zhou^1,2,3^, Lijun Wang^4^,*, Ning Zhuo^4^, Junqi Liu^4^, Jinchuan Zhang^4^, Shenqiang Zhai^4^, Shuman Liu^4^, Fengqi Liu^4,1^, & Quanyong Lu^1^,*

*Correspondence: Quanyong Lu [(luqy@baqis.ac.cn](mailto:(luqy@baqis.ac.cn)) or Lijun Wang ( [ljwang@semi.ac.cn](mailto:ljwang@semi.ac.cn))

^1^Division of Quantum Materials and Devices, Beijing Academy of Quantum Information Sciences, Beijing 100193, China;

^2^Beijing National Laboratory for Condensed Matter Physics, Institute of Physics, Chinese Academy of Sciences, Beijing 100190, China;

^3^School of Physical Sciences, University of Chinese Academy of Sciences, Beijing 100049, China;

^4^Key Laboratory of Semiconductor Materials Science, Institute of Semiconductors, Chinese Academy of Sciences, Beijing 100083, China;

^5^These authors contributed equally to this work.

**1. Emission characteristics of conventional ridge lasers**

The quantum cascade laser (QCL) region is based on a bound-to-bound (BTB) optical transition hybridized with a miniband transportation and phonon-assisted depopulation scheme^[1]^. The intersubband transition process is shown in Figure S1a. The QCL active region core is designed with layer thickness in angstrom of 48/90/35/103/11/98/52/172 targeting a frequency of 3.9 THz. The 12.15-μm thick QCL active region is sandwiched between two metallic cladding layers to create a double-metal waveguide with an almost unitary confinement factor. Figure S1b show the QCL active region gain spectrum peaks around 3.9 THz, where the bias voltage is 56 mV/module. A FP device with a cavity length of 1.7 mm and a ridge width of 80 μm shows output power of 4 mW at 13 K with a slope efficiency of 35 mW/A, as shown in Fig. S1c. The device exhibits a low threshold current density 108 A/cm^2^ due the strong gain active design and a low doping level in the active region^[1]^. The inset in Fig. S1c is the lasing spectrum of the FP device at 1.2 I_th_ at 13 K.


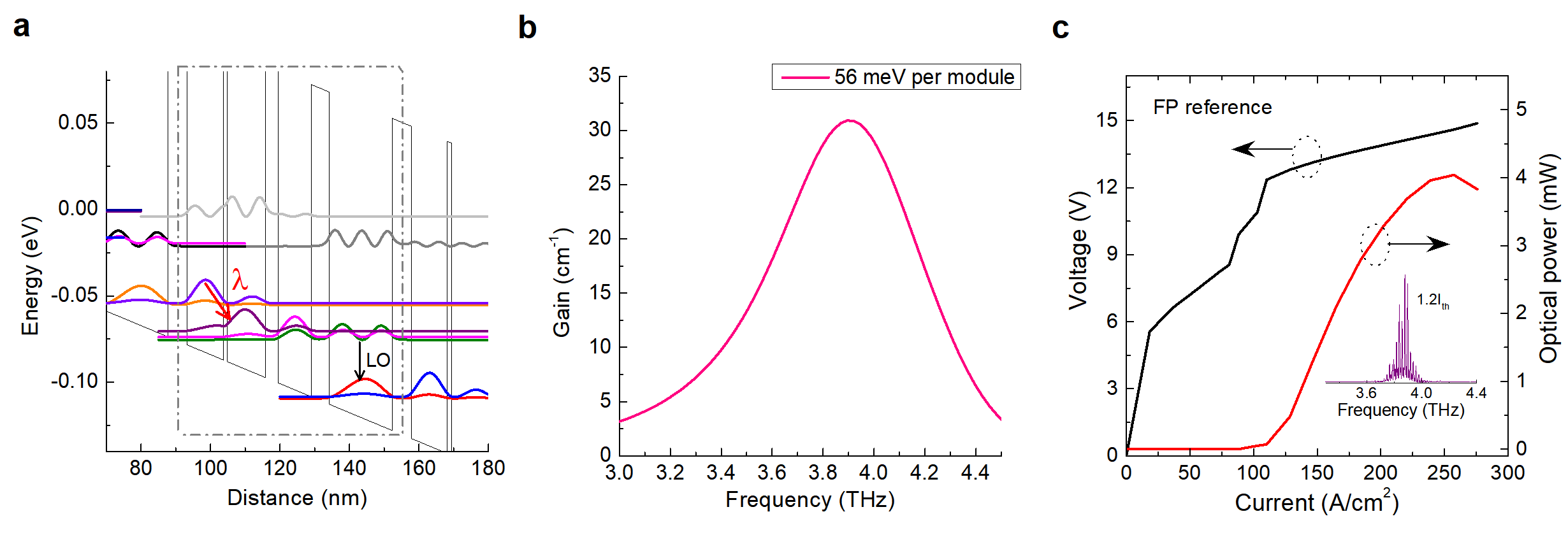


**Figure S1**. Conventional ridge laser has been fabricated and characterized. **a** Conduction band diagram of a THz QCL with four quantum wells per module in the active region. **b** The gain peak is at approximately 3.9 THz. **c** P-I-V characterization of a 1.7-mm long, 80-μm wide FP device at 13 K. Inset: lasing spectrum of the FP device at 1.2 I_th_ at 13 K.

1. **Phase-engineered photonic-crystal resonators at different shifts**

Figure S2a presents a schematic of a phase-engineered photonic-crystal (PEPC) lattice, which is composed of two sets of lattice groups (indicated in ellipse and circle), the circle lattice is shifted in the *x* and *y* directions by *d* from the ellipse points (*a* is the lattice constant and is approximately equal to the wavelength in the material, *λ*). PEPC resonator at *d=*0.3*a* is used as an example to illustrate the construction of a PEPC lattice. This unique PEPC structure produces the THz wave resonance effect in a broad area, which is not observed in conventional single-lattice structures, as shown in the following. As a result of the couplings among the fundamental Bloch waves, the eigensolutions of the PEPC structure become four band-edge modes at the *Γ_2_* point: mode A, B, C, and D. Figure S2b shown the Transverse-Magnetic (TM) band structure around the *Γ_2_*-point band edges of a typical PEPC, as indicated by the red circle. Here, the band of the PEPC is obtained by using the equivalent 2D COMSOL simulation. The electric field patterns of the four band-edge modes in a PEPC are shown in Figure S2c, where the color-map indicates the out-of-plane component *E_z_*, and the vector map exhibits the in-plane components *E_x_* and *E_y_*. The normalized mode frequencies and the vertical losses of the PEPC band-edge modes are obtained by using the three-dimensional full wave simulations via finite element method based on the commercial software of COMSOL Multiphysics, as shown in Figure S2d. Periodic boundary conditions are adopted in the simulation, which means the in-plane energy leakage is not considered. The vertical losses are defined as the PEPC resonators losses of the eigenmodes in the simulation: $\alpha=2\pi/(aQ)$, where $a$ is the lattice constant of the PEPC supercell, and Q is the quality factor. At the shift of *d =* 0.3*a*, the optical overlap factors with the active region ($\Gamma=\int_{Act} \left| E_{z} \right|^{2}dV/\int_{All} \left| E_{z} \right|^{2}dV$) for modes A, B, C and D are 99.943%, 99.809%, 99.566% and 99.218%, respectively, It can be seen that they have almost the same overlap factor. Due to the high vertical loss, mode A is unlikely to be the lasing mode. Mode D instead, with the lowest loss, would be the lasing candidate.


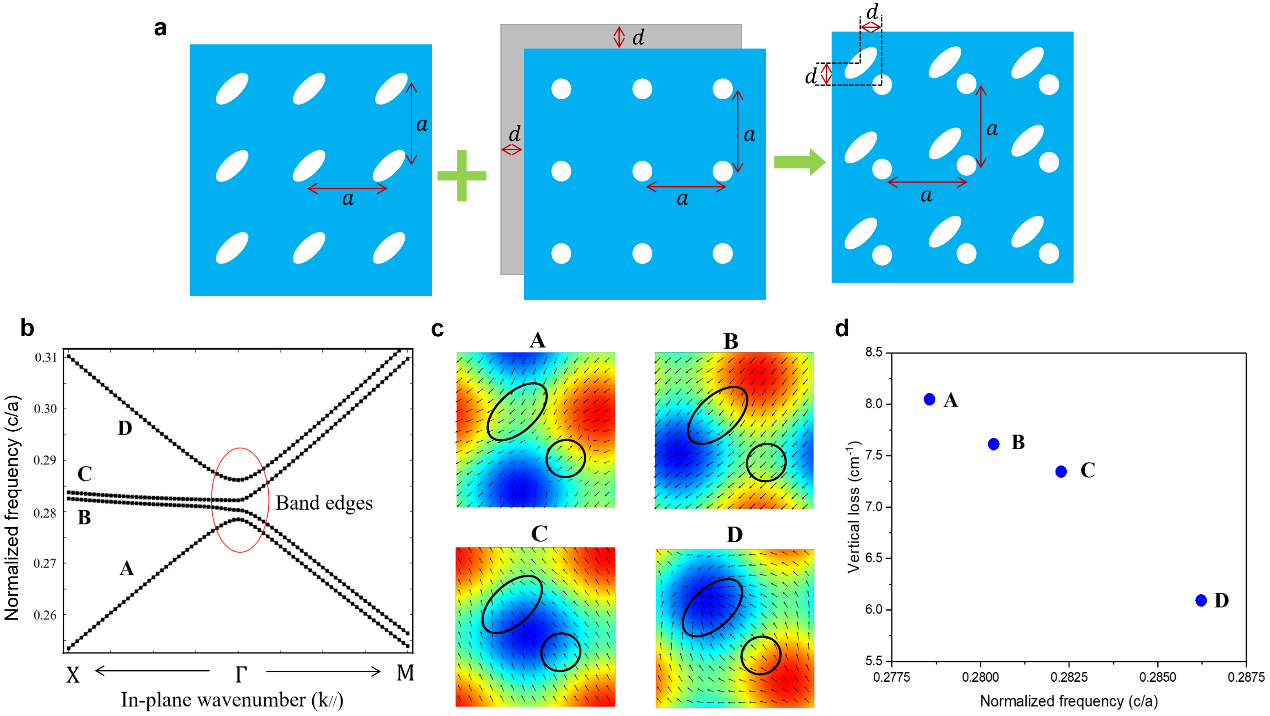


**Figure S2. Construction of THz PEPC and Photonic band structure (TM) diagram. a** Construction of PEPC resonators by shifting the circular-unit-cell lattice with respect to the elliptical-unit-cell lattice by a phase shift *d* in *x* and *y* directions; **b** Band structure of a PEPC for Transverse-Magnetic (TM) modes. An enlarged image of this band structure around the *Γ_2_*-point band edges, whose edges of bands A~D are circled in red. **c** The mode patterns in a PEPC of the four band-edge modes at the *Γ_2_* point of the THz PEPC QCL. The color-map indicates the out-of-plane component *Ez*, and the vector map indicates the in-plane electric field components *Ex* and *Ey*. **d** Modal vertical loss of PEPC lattice as a function of modal frequency. The period of the PEPC is 21.5 µm in the simulation.

1. **Brightness of surface emitting THz PEPC QCL beams**

The brightness *B* of a terahertz quantum cascade laser (QCL) is given by^[2]^:

$B=\frac{P_{out}}{S\times\Omega}=\frac{P_{out}}{\left(\text{M}^{2}\right)^{2}\lambda^{2}}$ (S1)

where *P_out_*, *S*, *Ω,* λ and M^2^ are the output power, emission area, solid angle, wavelength and beam quality factor of the THz surface emission QCL, respectively. The beam quality factor M^2^ is calculated according to $M^{2}=4\pi\sigma_{NF}\sigma_{FF}/\lambda$, where $\sigma_{NF}$and $\sigma_{FF}$are the standard deviations of the calculated near field profile of the fundamental lateral mode for a 1.6×1.6 mm^2^ cavity and the measured far field profile, respectively^[3]^. According to Eq.(S1), higher brightness can be achieved by increasing the output power *P_out_* while keeping the product of the emission area *S* and the beam divergence *Ω* as small as possible^[4]^. Ideally, for a THz QCL with a single (lateral) mode, the output power *P_out_* is proportional to the emission area *S*, and increasing *S* narrows the solid angle *Ω* of the emitted beam. Altogether, *P*∝*S* and Ω∝1/*S*. For our surface-emitting THz PEPC QCL, the output power at 6A is 185mW, which translates to a brightness value B=1.6×10^7^ Wsr^−1^m^−2^, according to the measured M^2^=1.4. The measured output optical power and beam quality of the THz PEPC QCL were used to determine the optical brightness from Eq. (S1).

Theoretically, our surface-emitting PEPC QCL can achieve even higher brightness by increasing the size of the PEPC or by optimizing the spacing between the elliptical air hole and the circular air hole of the PEPC. Figure S3a shows the PEPC supercell, which is composed of elliptical and circular lattice points with a shift of *d~*0.25*a* along the *x* and *y* directions. According to the 3D coupled wave theory^[5-8]^, it can be concluded that the phase-engineered photonic crystal resonant cavity is the most advantageous when *d~*0.25*a*, owing to this (1/4)*λ* shift, a (1/2)*λ* optical path difference exists between every two adjacent lattice points (see the red arrows in Figure S3a, resulting in the destructive interference of the back-diffracted light waves. This is in striking contrast to the widely used π phase shift (corresponds to *d*=0.5*a*) in a second-order grating, which results in a constructive interference^[9]^. The PEPC design in this work is a kind of global 2D extension of phase shift *d* in both *x* and *y* directions. By using different shapes of unit cells and a proper phase shift *d*, desired coupling is achieved to enable mode profile flattening over the entire device area. On the other hand, the (1/2)*λ* optical path difference does not exist for light waves diffracted 90°, so a similar cancellation does not occur in these directions. In this way, the PEPC spreads out the overall light distribution while continues acts a resonator via the preservation of 2D optical coupling. Actually, the light waves propagating inside a periodic metallic PEPC resonators include a large number of wave-vector components and they interact with each other in a more complex manner, Figure S3b illustrates the out-of-plane coupling between the basic waves and radiative waves (red arrow) that is induced by first-order Bragg diffraction.


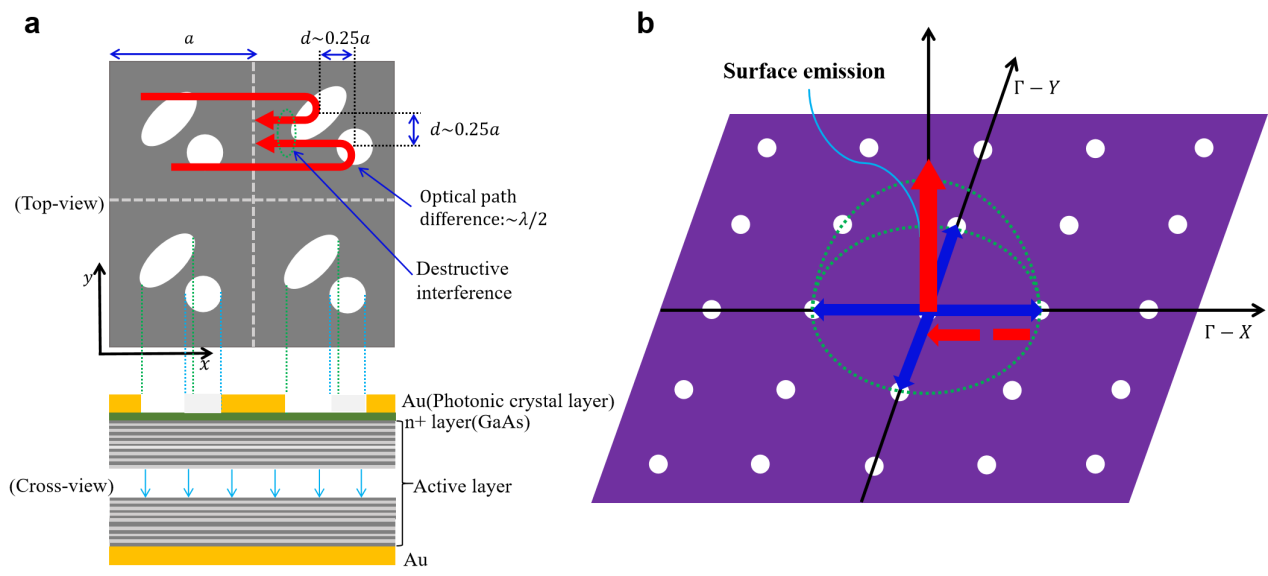


**Figure S3. Schematic of optical coupling inside the PEPC lattice. a** Schematic of in-plane destructive interference for 1D coupling inside a photonic-crystal resonator with *d~*0.25*a* and its cross-section view; **b** Schematic of out-of-plane coupling due to first order Bragg diffraction in a phase-engineered photonic crystal (blue arrow).

1. **Modelling of a PC QCL**

Figures S4a-c show the electric-field distributions of the fundamental mode, first high-order mode, second high-order mode for a PC QCL. For a fair comparison, the PC structure has the same filling factor and lattice constant as these of PEPC, as shown in Figure S4d. Different cavity modes have different mode losses, where the fundamental mode has the lowest loss, and the first and second high-order modes have lower quality factors (or higher losses). Figure S4e shows the In-Plane loss margins between the fundamental mode and first higher-order mode, and second higher-order mode of the PC resonator as a function of different device area. When the size *L* of the PC resonator increases, the losses of the fundamental and high-order modes are converging to the same level. This means decent loss margins between the fundamental and higher-order modes can only be obtained by decreasing the device area, which is opposite to the strategy of beam brightness upscaling with device area. In addition, for a PC QCL and PEPC QCL (*d=*0.3*a*) with the same area of 1.3×1.3 mm^2^, the calculated out-plane loss of PEPC QCL is almost twice that of PC QCL, this means twice the output optical power for PEPC QCL over PC QCL.


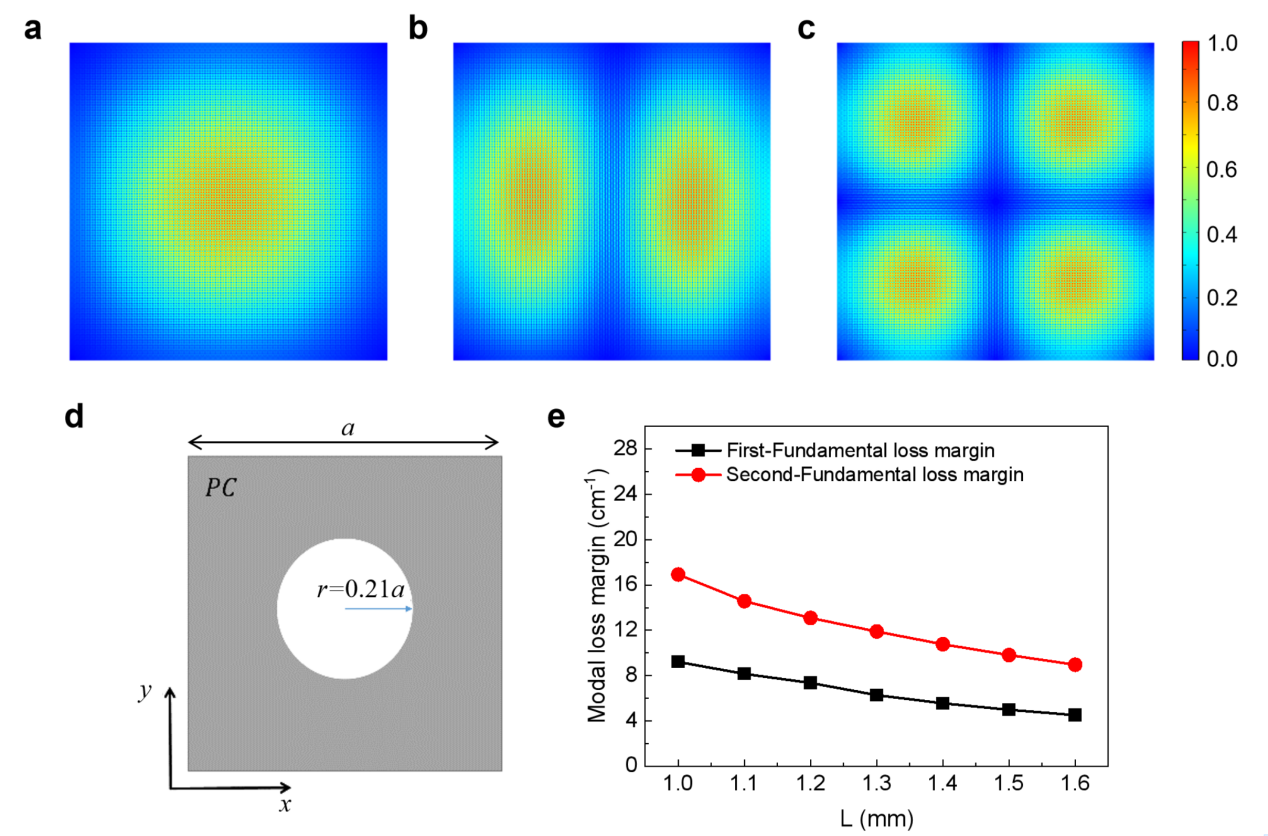


**Figure S4. Electric-field distributions and in-plane loss margins of eigenmodes. a-c** Calculated electric-field distributions of the fundamental mode, first high-order mode, second high-order mode in a PC QCL with *L*=1.3 mm; **d** Schematic of a unit cell of the PC lattice. **e** Modal loss margins between the fundamental and first high-order modes, and fundamental and second high-order modes in PC resonator as a function of the device size *L*.

1. **Output power of** **THz PEPC QCLs at different shifts**

Based on the same QCL wafer, we also investigated the influence of shift *d* over the output power for the PEPC QCLs. In the experiment, and we fabricated and tested THz PEPC QCLs with different shift *d* values, while keeping the rest parameters the same for comparison. As shown in Fig. S5a, the power of the devices with *d=*0.3*a* has the maximum output optical power (over 185 mW). Theoretically, as *d* decreases to 0.25*a*, the out of plane radiation loss will increase when sufficient in-plane coupling is provided. For the device area used in this work, the in-plane optical feedback is under coupled when *d=*0.25*a*, which leads to greater in-plane losses and lower output power as shown in Fig. S5a. Consider the coupling coefficients showing in Fig. 2d for *d*=0.25*a*, the device size needs to be greater than 3.0×3.0 mm^2^ for stable single-mode lasing operation with an optimal output efficiency, which would be our future work. To better comprehend how the optimal shift condition is determined for the PEPC device with *L=*1.6 mm, we calculated a figure of merit Δ*α*/*α*_0_ and plotted as a function of shift. Here, α_0_ is the in-plane loss of the fundamental mode and Δ*α* is the loss margin of fundamental mode and first high-order mode. As shown in Fig. S5b, while the loss of the fundamental mode goes up rapidly for *d*<0.3*a*, the figure of merit Δα/α_0_ peaks at *d=*0.3*a*, as shown in Fig. S5c, which indicates the optimal combination in terms of the loss margin and the loss for the fundamental mode. For devices with larger areas, similar procedure can be performed to determine the optimal shift parameter.


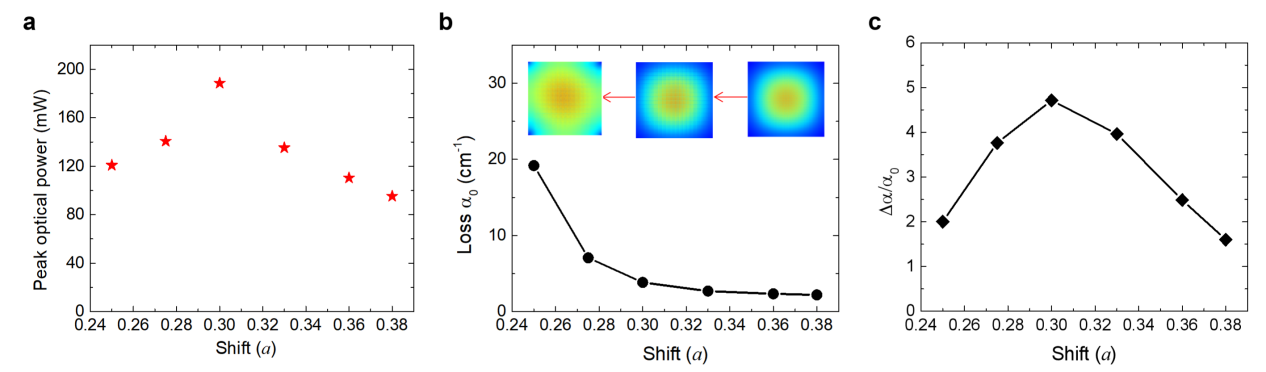


**Figure S5**. **THz PEPC QCLs at different shifts. a** The experimental output power of surface-emitting THz PEPC QCLs at different shifts with *L*=1.6 mm. **b** Calculated in-plane loss α_0_ of the fundamental mode as a function of phase shift. **c** The figure of merit Δ*α*/*α*_0_ as a function of phase shift.

1. **Power performance comparison between different devices sizes and lattices**

Based on the same QCL wafer, we also manufactured devices with different sizes of 1.3×1.3 and 1.6×1.6 mm^2^ with adjusted shifts *d* = 0.36*a* and 0.3*a* correspondingly for the brightness upscaling experiment. As shown in the Figure S6, the power of the device with *L*=1.6 mm is much higher than that of device with *L*=1.3 mm. Therefore, if the size of the device is further increased, the output power can be further improved. Furthermore, to confirm that our PEPC resonators has a better out-plane radiation characteristics, we also fabricated a regular square single lattice photonic crystal surface emission QCL with the same filling factor as the PEPC. As shown in the Figure S6a, under the same device size and the same QCL wafer, the PEPC surface emitting QCL has more than twice the output power of a regular square lattice photonic crystal surface emitting QCL. Figure S6b show the experiment far-field emission pattern for the THz PEPC QCL with *L=*1.3 mm, and the far-field emission pattern is primarily a single-lobed distribution. Figure S6c show the experiment far-field emission pattern for the regular square single lattice PC surface emission QCL with the same device area. Multi-lobed far field is observed due to the much smaller modal loss margin as discussed above.


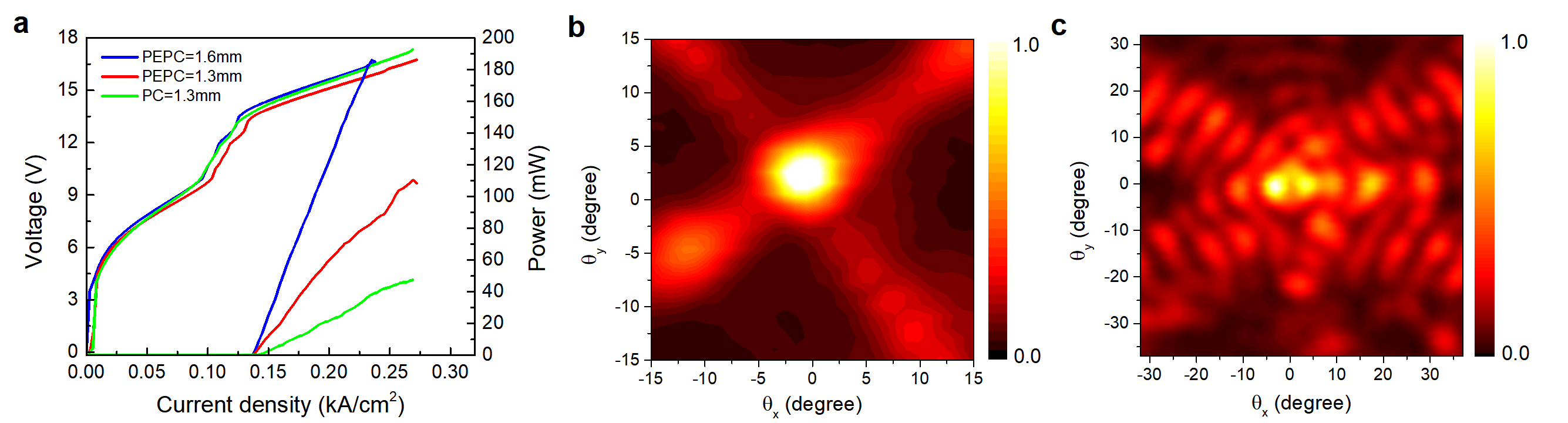


**Figure S6.** **Comparison of power performance of devices with different device size and different lattices.** **a** PIV curves of PEPC QCLs with *L*=1.6 mm, *L*=1.3 mm, and a PC QCL device with *L=*1.3 mm. **b-c** Far-fields measured from the PEPC QCL and PC QCL with the same filling factor and device area.

### Reference

1. Li, W., Li, Y., Ma, Y. et al. Continuous-wave terahertz quantum cascade laser based on a hybrid bound to bound quantum design. Frontiers in Photonics 3, 1071879 (2022).
2. Naidoo, D., Litvin, I. A., Forbes, A. Brightness enhancement in a solid-state laser by mode transformation. Optica 5, 836-843 (2018).
3. Heydari, D., Bai, Y., Bandyopadhyay, N. et al. High brightness angled cavity quantum cascade lasers. Applied Physics Letters 106, 091105 (2015).
4. Yoshida, M., De Zoysa, M., Ishizaki, K. et al. Double-lattice photonic-crystal resonators enabling high-brightness semiconductor lasers with symmetric narrow-divergence beams. Nature Mater 18, 121–128 (2019).
5. Liang, Y., Peng, C., Sakai, K. et al. Three-dimensional coupled-wave analysis for square-lattice photonic crystal surface emitting lasers with transverse-electric polarization: finite-size effects. Opt. Express 20, 15945-15961 (2012).
6. Peng, C., Liang, Y., Sakai, K. et al. Three-dimensional coupled-wave theory analysis of a centered-rectangular lattice photonic crystal laser with a transverse-electric-like mode. Phys. Rev. B 86, 035108 (2012).
7. Liang, Y., Peng, C., Ishizaki, K. et al. Three-dimensional coupled-wave analysis for triangular-lattice photonic-crystal surface-emitting lasers with transverse-electric polarization. Opt. Express 21, 565-580 (2013).
8. Yang, Y., Peng, C., Liang, Y. et al. Three-dimensional coupled-wave theory for the guided mode resonance in photonic crystal slabs: TM-like polarization. Opt. Lett. 39, 4498-4501 (2014).

[9] Kumar, S., Williams, B. S., Qin, Q. et al. Surface-emitting distributed feedback terahertz quantum-cascade lasers in metal-metal waveguides. Opt. Express 15, 113-128 (2007).
